# Maternal instincts: a review of the role of chemosensory cues and larval habitat conditions on oviposition site choice by gravid *Aedes aegypti* (Diptera: Culicidae)

**DOI:** 10.1093/jme/tjag107

**Published:** 2026-07-01

**Authors:** Nicole S Rodrigues, Geoffrey M Attardo

**Affiliations:** Department of Entomology and Nematology, University of California Davis, Davis, CA, USA; Department of Entomology and Nematology, University of California Davis, Davis, CA, USA

**Keywords:** *Aedes aegypti*, oviposition, behavior, breeding site, geosmin

## Abstract

*Aedes aegypti* (*Ae. aegypti*) is a global vector of arboviruses, including those causing dengue, chikungunya, Zika, and yellow fever. Effective management of *Ae. aegypti* populations remain challenging due to their anthropophilic feeding behavior, their exploitation of urban environments, their complex oviposition behavior, and their egg physiology. The characteristics of larval habitats can influence various aspects of larval development, thereby altering adult physiology and behavior. These include differences in blood-feeding behavior, reproductive capacity, and lifespan. Gravid females select oviposition sites based on a diverse array of visual, textural, chemical, and biological cues that attract/repel females from sites and/or stimulate/inhibit oviposition, including volatile organic compounds from microorganisms, detritus, conspecific/heterospecific larvae and adults, predator activity, and environmental factors. This review focuses on the chemosensory aspects of oviposition site selection. Understanding the effects of concentration-dependent chemical cues on oviposition behavior in gravid female *Ae. aegypti* offers critical insights into the ecology of this species. Integrating this knowledge into vector control strategies, particularly attract-and-kill ovitraps incorporating optimized attractants and larvicides, could enhance surveillance efficacy and reduce *Ae. aegypti* populations. As climates become more favorable and urbanization expands, *Ae. aegypti*’s geographic range is likely to expand, making it essential to leverage detailed behavioral and ecological insights to improve surveillance, vector management, and to mitigate arboviral disease risks globally.

## Graphical abstract

**Graphical Abstract:**
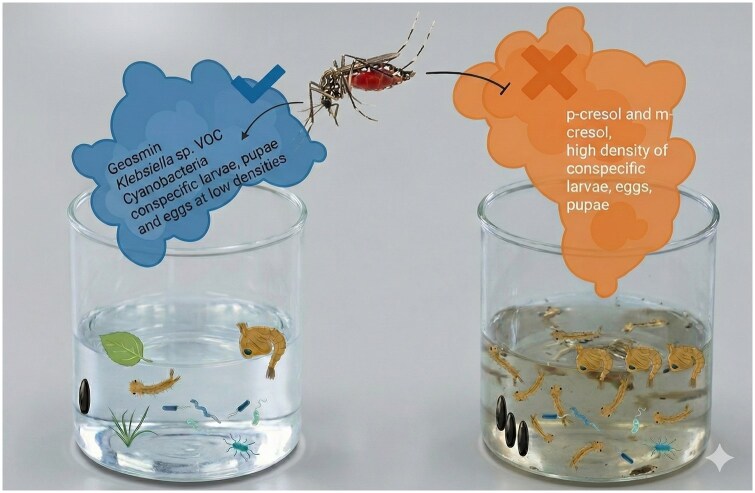

## Introduction

*Ae. aegypti* (L.) is a successful invasive vector due to its adaptations to urban environments and its eggs’ ability to survive desiccation (OECD 2018). This urban–suburban mosquito is commonly known as the yellow fever mosquito. *Ae. aegypti* is a primary vector of numerous medically important arboviruses, including yellow fever, dengue, chikungunya, and Zika (OECD 2018, Carvajal-Lago et al. 2021, Egid et al. 2022, Mosquera et al. 2023). These diseases are a growing global concern with an expanding distribution and increasing incidence among human populations (Wang et al. 2019, Egid et al. 2022). Vector control-based strategies are among the most effective means of preventing vector-borne diseases (Carvajal-Lago et al. 2021). Historically, *Ae. aegypti* was a forest-dwelling mosquito that bred in tree holes across sub-Saharan Africa. Approximately 5 centuries ago, global trade and human movement facilitated its spread beyond its native range, enabling it to invade new regions across the globe (Powell and Tabachnick 2013, Powell et al. 2018). Today, *Ae. aegypti* has 2 distinct subpopulations: *Ae. aegypti formosus*, the ancestral form still found in African forests, and *Ae. aegypti aegypti*, which has adapted to urban environments (OECD 2018). Female *Ae. aegypti formosus* prefer non-human blood meals for egg production and lay their eggs in coconut husks or tree holes (OECD 2018, Pruszynski et al. 2020).

For this review, references to *Ae. aegypti* will specifically pertain to *Ae. aegypti aegypti*, the urban-adapted form. This urban mosquito species is distributed between 40°N and 40°S latitude.

Although females strongly prefer human hosts, they will feed on other vertebrates if necessary (Gulia-Nuss et al. 2015, Pruszynski et al. 2020, Egid et al. 2022). Gravid (egg-carrying) females use chemosensory (eg olfactory) cues (eg chemicals produced by microbes) as well as visual, gustatory, and tactile cues to select an oviposition site (Afify and Galizia 2015). They preferentially deposit eggs in artificial containers found in urban environments, including discarded trash, tires, flower pots, and other water-holding items, which make up an estimated 95% of oviposition sites (OECD 2018, Wilke et al. 2019, McGregor and Connelly 2021, Walker et al. 2021, Egid et al. 2022). In field and lab experiments, black ovitraps have more eggs deposited than blue, green and transparent ovitraps (Khan et al. 2022). Human infrastructure and behavior often provide conditions that support *Ae. aegypti* breeding including sewage treatment plants, storm drains, and irrigated lawns (Wilke et al. 2019). Improved public outreach and communication about this would enable the public to be aware of the disease risks and lessen the risks of misinformation (Thomas et al. 2024).

*Ae. aegypti* eggs, typically ∼1 mm in length and ovoid in shape, are white when freshly laid and turn black after a few hours (OECD 2018). The eggs are resistant to desiccation and capable of surviving for months until rehydration triggers hatching. Depending on resource availability, larvae can develop into adults in as little as a week or as long as 90 d (Walker et al. 2021). Their diet consists primarily of detritus, microbes, and small invertebrates, from which they derive essential nutrients required for growth, including sugars, salts, lipids, amino acids, and vitamins (Singh and Brown 1957).

Over time research has made strides into examining factors influencing oviposition by mosquitoes. Oviposition site selection is a critical decision gravid females make to give their offspring the best chance at survival, as the immobile larvae cannot move to new aquatic environments if resources become scarce or conditions deteriorate. Site selection can be influenced by olfactory cues emitted from aquatic habitats. These cues that draw gravid females to potential oviposition sites are called oviposition attractants (Afify and Galizia 2015, Khan et al. 2023). While an oviposition repellent is an olfactory cue that repels gravid females from an oviposition site. An oviposition stimulant is a chemosensory (not necessarily olfaction) cue that elicits oviposition behavior once at a site. The opposite of a stimulant is a deterrent, a chemosensory cue that deters oviposition. Attractants and repellents work from long distances from the oviposition site while stimulants and deterrents work closer to the oviposition site.

After choosing an oviposition site, to create a more supportive larval environment, *Ae. aegypti* can shape the microbial community as oviposition causes a decrease in microbial alpha diversity (Mosquera et al. 2023). After ovipositing, gravid *Ae. aegypti* may also defecate, introducing beneficial microbes to the breeding site (Mosquera et al. 2021). For instance, *Ae. aegypti* can inoculate the bacteria *Elizabethkingia* into the oviposition site (Mosquera et al. 2023). The presence of these microbes can support larval health and development (Mosquera et al. 2023).

Temperature also plays a key role in development and behavior. The viable range for *Ae. aegypti* is approximately from 14 °C to 30 °C (OECD 2018). At 25 °C, development from egg to adult may take slightly over a week (OECD 2018). Lower temperatures slow development and can extend the larval period significantly (eg months), while higher temperatures enable larval growth, development, and movement. In adults, higher temperatures compared to lower ones reduce their lifespans (OECD 2018). The progression of climate change is resulting in new geographic regions, such as parts of the United States, that are becoming more hospitable to *Ae. aegypti* (Reinhold et al. 2018, CDC 2020, Walker et al. 2021, CDC 2024, Abbasi 2025, Heath et al. 2025, Zhang et al. 2025). The ability of this species to successfully overwinter in new locations has allowed the mosquito to expand its range, as seen in California, where *Ae. aegypti* arrived in the summer of 2013, likely following multiple undetected introductions and has continued to spread through the state (Gloria-Soria et al. 2014, Pless et al. 2017, VectorSurv Maps).

Aquatic insects are classic bioindicators used to monitor the quality of underwater environments (Fadel et al. 2023). Because they depend on water for much of their development, changes in their presence, density, and behavior can reflect subtle environmental changes, such as variations in nutrient availability, pollution, or habitat disturbance. This principle not only applies to ecologically important species but also underscores the broader relevance of understanding *Ae. aegypti* oviposition behavior in relation to environmental health. For instance, *Ae. aegypti* larval density can be correlated to differences in water conductivity, pH, ammonium, and phosphate levels (Avramov et al. 2024).

Oviposition site selection by *Ae. aegypti* is influenced by a complex array of chemical signals, including bacterial volatiles, conspecific byproducts, and physical attributes such as container size, color and water quality (Boullis et al. 2021, Khan et al. 2022, 2023, Prasad et al. 2023). The primary focus of this review will be on chemical stimuli of mosquito oviposition behavior. Experiments have demonstrated that gravid females use these cues to evaluate potential egg-deposition sites, balancing the risks of predation, competition, and habitat suitability (Albeny-Simões et al. 2014, Mosquera et al. 2021). This review aims to synthesize current knowledge on *Ae. aegypti* oviposition behavior as well as the chemical and biological factors that influence egg-laying decisions.

Due to their strong preference for urban environments and human-derived habitats for reproduction, *Ae. aegypti* exploit a wide array of breeding sites created by human activities, making their populations difficult to control and monitor (Wilke et al. 2019, Prasad et al. 2023). Traps can help reduce *Ae. aegypti* populations, however source reduction is needed prior to trap deployment (Perich et al. 2003, Eiras et al. 2021, Khan et al. 2022). Understanding these processes and gaining a deeper understanding of site selection will aid the development of cheaper, novel and lethal oviposition traps, and targeted interventions to control and monitor *Ae. aegypti* populations.

## Oviposition Behavior in *Ae. aegypti*

*Ae. aegypti* females exhibit complex oviposition behavior driven by ecological pressures and reproductive biology. A key strategy is skip oviposition, where a female distributes her eggs across multiple sites within a single gonotrophic cycle (Craig et al. 2008, Powell et al. 2018, Walker et al. 2021). This behavior minimizes the risk of larval overcrowding and competition for limited resources such as food and space, thereby improving the survival chances of her offspring (Craig et al. 2008, Walker et al. 2021). One study using oviposition traps in *Ae. aegypti* endemic areas found that around 77% of ovitraps contained fewer than 30 eggs, with each gravid female typically laying 11 to 30 eggs per site (Chadee 2009).

Another noteworthy behavior is superoviposition, in which females lay eggs at sites that already contain conspecific eggs or larvae (Ganesan et al. 2006, Craig et al. 2008). Field and laboratory studies have shown that superoviposition tends to occur when egg densities at a site are low, typically fewer than 25 to 30 eggs (Craig et al. 2008, Chadee 2009). While skip oviposition generally favors unoccupied sites, low densities of existing eggs or larvae may actually encourage additional oviposition (Chadee 2009, Khan et al. 2023). The presence of conspecific eggs or larvae at low densities can signal a resource-rich environment, this can stimulate egg-laying, whereas sites that are heavily contaminated or exhibit signs of high larval densities act as oviposition deterrents due to resource competition (Chadee 2009, Gonzalez et al. 2016, Shragai et al. 2019, Boullis et al. 2021, Mosquera et al. 2021, Khan et al. 2023). This behavior is seen in *Aedes albopictus* (*Ae. albopictus)* as well.

While laboratory experiments have shown that when only a single oviposition site is available, even one with a high egg density, females are unlikely to retain their eggs and will lay them there (Craig et al. 2008). However, when multiple sites are available, skip oviposition dominates (Craig et al. 2008). Older eggs may also be less effective at deterring further oviposition, possibly because egg-associated chemical markers fade over time (Chadee 2009).

The presence of late-stage larvae (third- and fourth-instar) also stimulate oviposition in both *Ae. aegypti* and *Ae. albopictus*, likely because their development indicates a productive and resource-rich site (Boullis et al. 2021, Mosquera et al. 2021, Khan et al. 2023). In an oviposition choice assay, females were offered a control site containing larval food in water alongside experimental sites containing either eggs frozen at −20 °C for 30 min, second- or fourth-instar larvae, or pupae and pupal exuviae. Females preferentially oviposited in sites containing fourth-instar larvae (Khan et al. 2023).

*Ae. albopictus* and *Ae. aegypti* larvae can be found coexisting in the same habitats, such as artificial containers (Gonzalez et al. 2016). The larvae of both species are very competitive during the larval stage (Gonzalez et al. 2016). When *Ae. albopictus* has the choice of breeding sites with no larvae, breeding sites of conspecific (same species, ie *Ae. albopictus*) or heterospecific (different species, ie *Ae. aegypti*) larvae, they chose the breeding sites with conspecific/heterospecific larvae (Shragai et al. 2019, Tuñón et al. 2025). This preference is influenced by both the species and density of larvae present. Conspecific larvae are generally attractive for oviposition, but the response is density-dependent: moderate densities (eg 100 larvae per 3.7 L) promote oviposition, whereas very high densities (eg more than 130 larvae per 0.37 L) act as a deterrent (Shragai et al. 2019). In sites containing between 10 and 100 heterospecific larvae, no oviposition effect was observed for either *Ae. aegypti* or *Ae. albopictus* (Gonzalez et al. 2016). In an experiment when conspecific and heterospecific larvae were removed, *Ae. aegypti* did not avoid heterospecific breeding sites for oviposition—this may indicate the visual cue of larvae enables *Ae. aegypti* to detect other species (Tuñón et al. 2025). These dynamics reflect the delicate balance that gravid females strike between resource availability and competitive pressures.

Not only do gravid *Ae. aegypti* use conspecific immatures in breeding sites as cues for oviposition but they also use other conspecific female mosquitoes as cues. Aggregations of female *Ae. aegypti* at breeding sites can occur and females use chemosensory dependent communal cues to regulate their population densities to either aggregate or disperse (Costa-da-Silva et al. 2024). Aggregation occurs independently of oviposition activity, water interaction and skip oviposition. Lower densities of aggregation may indicate a suitable breeding site. There may be as of yet unidentified intraspecific adult VOCs that enable adult female mosquitoes to locate each other. Understanding these nuanced behaviors is essential for developing effective vector control strategies. By leveraging the tendency of gravid *Ae. aegypti* to respond to specific environmental signals and their avoidance of overcrowded sites; targeted interventions, such as oviposition traps, habitat management, and lures that aggregate female mosquitoes could be designed to reduce ­mosquito population growth and disease transmission.

## Chemical Cues and Predators in Oviposition Site Selection

It is crucial for gravid female *Ae. aegypti* to choose sites that provide the best chance for survival and optimal development for their offspring. Factors such as the presence of nutrients, reduced competition from conspecifics as well as heterospecifics, and an absence of predators optimizes the chances for proper development and survival of offspring (Mosquera et al. 2021, Khan et al. 2022, 2023). Site selection is in part influenced by a complex interplay of chemical cues in the environment, which signal the availability of resources, the risk of competition or predation, and the overall suitability of the habitat for offspring survival. Physicochemical properties such as conductivity, total dissolved solids, and water hardness are important factors for oviposition (Motta-Yanac et al. 2025). Polarized light can also enable mosquitoes to detect aspects of the breeding site such as pH but they use other senses, such as touch, to detect suitable habitats. Prior to oviposition, gravid females will touch the water of a site with their legs and mouthparts. Experiments removing different sensory organs of the mosquito found that chemoreceptors on the tarsi and potentially tibia are required for perception of compounds that act as oviposition stimulants or deterrents (Hudson 1956).

For instance, high salt concentrations are lethal to *Ae. aegypti* larvae, so gravid females will avoid ovipositing in sites with high levels of sodium (Hudson 1956, Matthews et al. 2019). Experiments showed that they can detect the difference between 0 and 0.136 M of NaCl in water with fewer eggs laid in solutions at or above 0.085 M NaCl (Hudson 1956). Additional experiments demonstrated that high concentrations of KCl, MgCl_2_ and Na_2_SO_4_ have similar effects on oviposition (Hudson 1956). Certain sensory neurons express ppk301, a member of the DEG/ENaC (degenerin/epithelial sodium channel) family, and play a key role in initiating oviposition (Matthews et al. 2019). These neurons exhibit 2 distinct responses: a ppk301-dependent response to water that drives gravid females to oviposit in sites with low salt concentrations, and a ppk301-independent response to salt that inhibits ­oviposition under high-salt conditions (Matthews et al. 2019).

CO_2_ also appears to function as an oviposition cue. It is a well-known cue for host seeking behavior. However, the function of CO_2_ changes in gravid female mosquitoes with CO_2_ levels enabling female *Ae. aegypti to* either aggregate or disperse at breeding sites (Costa-da-Silva et al. 2024). High CO_2_ levels can act as a communal cue to signal dispersion thereby preventing high densities of female mosquitoes aggregating around a single breeding site, thus preventing competition among offspring. This dispersal cue may also result from the presence of high densities of conspecific immatures or hosts or predators. CO_2_ levels do not differ between *Ae. albopictus* and *Ae. aegypti* larvae and does not appear to act as a specific cue of conspecific or heterospecific larvae in a site (Tuñón et al. 2025).

The presence of mosquito larvae is not the only biological factor considered during breeding site selection. Predator presence also influences oviposition site selection. For example, *Toxorhynchites (Tx.)* larvae, known predators of mosquito larvae, can be found in containers where *Ae. aegypti* lay their eggs. All instars of their larvae feed on *Ae. aegypti* larvae and they can be cannibalistic as well (Collins and Blackwell 2000). Prior to pupation *Tx.* fourth-instar larvae will kill other organisms in the breeding site, leaving the corpses of their prey uneaten. This is thought to be a defensive behavior to eliminate organisms that could threaten the survival of the vulnerable pupal stage (Collins and Blackwell 2000). One might expect these predators to deter oviposition; however, this behavior makes aquatic habitats more attractive for oviposition. The predatory activity results in reduced competition in aquatic habitats by consumption of early instar *Ae. aegypti.* Studies show that gravid *Ae. aegypti* females are not repelled by the presence of *Tx.* larvae in potential oviposition sites (Albeny-Simões et al. 2014). Predator-driven trophic cascades in small containers can indirectly increase microbial abundance and resource availability (Albeny-Simões et al. 2014). Their predatory activities reduce the density of competing larvae and can stimulate microbial growth through various mechanisms, such as partially eaten prey and fecal matter serving as substrates for microbial growth. Such effects are more pronounced in small aquatic habitats when only a few predators present. This creates conditions that favor egg-laying by gravid females, despite the predation risk (Albeny-Simões et al. 2014).

There are multiple reasons why *Ae. aegypti* have not evolved a strong preference for predator-free oviposition sites. Gravid females move more slowly, and the longer they spend searching for ideal sites increases the probability of predation by organisms such as spiders or dragonflies, prior to egg deposition (Albeny-Simões et al. 2014). Furthermore, their skip oviposition behavior increases the likelihood of offspring survival even in the presence of aquatic predation.

Overall, the ability of *Ae. aegypti* females to detect and respond to this complex array of chemical cues underscores the importance of these signals in shaping oviposition behavior. Understanding these factors can inform the development of targeted surveillance and control strategies, such as attractant-based oviposition traps and habitat management interventions.

## Volatile Organic Compounds and Other Oviposition Cues

Gravid *Ae. aegypti* females rely heavily on airborne and water-borne volatile organic compounds (VOCs) to evaluate potential oviposition sites. VOCs serve as evidence of microbial activity, of conspecific and heterospecific eggs and larvae, and of decaying organic matter, within aquatic habitats (Collins and Blackwell 2000, Mosquera et al. 2021, Khan et al. 2023). Gravid females use VOCs to assess site quality, balancing the benefits of nutrient-rich environments against the risks of overcrowding, cannibalism, or predation.

VOC cues from conspecific and heterospecific immature stages enable gravid *Ae. aegypti* to perceive the characteristics of a site. These include the presence (or absence) of site-specific resources, overcrowding from larval conspecifics/heterospecifics, sensation of the developmental stages of con/heterospecifics, and the water quality of the oviposition site. An analysis of cuticular hydrocarbons from eggs and first- to third-instar larvae demonstrated that those life stages exhibit similar chemical profiles after hexane extraction, followed by GC/MS analysis (Wang et al. 2019). Pupae do not appear to provide chemical signals that would influence females’ oviposition decisions (Khan et al. 2023). A subsequent experiment examined VOCs from each of those groups and treated the oviposition sites with multiple concentrations of a VOC blend in hexane as well as hexane alone. The study found that at high concentrations, fewer eggs were deposited, whereas at lower concentrations, oviposition was stimulated (Khan et al. 2023). As late-stage larvae transition into the non-feeding pupal stage, competition for resources decreases, however some resources may be reduced or depleted as a result of their prior development (Khan et al. 2023).

Infusion of potential oviposition sites with plants such as Bermuda grass can result in the release of p-cresol as a VOC (Afify and Galizia 2014). Laboratory assays have shown that low concentrations of p-cresol stimulate egg-laying behavior in *Ae. aegypti*, while higher concentrations (10^−8^ to 10³ ppm) deter it (Afify and Galizia 2014). Similar effects have been observed in other mosquito species, such as gravid *Culex (Cx.) quinquefasciatus* and *Cx. tarsalis*, which are attracted to p-cresol at 10^−4^ ppm. A concentration of 10^−1^ ppm stimulates egg-laying in *Cx. quinquefasciatus*. In 2 species of *Tx.* mosquitoes (*Tx. brevipalpis* and *Tx. amboinensis*), p-cresol at concentrations of 1, 10, and 50 ppm stimulates oviposition, while for *Tx. splendens*, 10 ppm is effective. In *Ae. albopictus*, p-cresol stimulates oviposition at 10^−5^ ppm, while 8.3 ppm acts as a repellent.

In contrast, m-cresol is largely inactive for *Ae. aegypti*, except at the highest dose tested, 10³ ppm, which acts as a deterrent. For other mosquito species, including *Ae. triseriatus*, *Tx. moctezuma*, and *Tx. amboinensis*, m-cresol at 3 ppm stimulates egg-deposition. When both p- and m-cresols are present together in a 1:1 mix, they act as an oviposition deterrent. When presented in separate cups within the same cage at 10^2^ ppm, both cresols resulted in significantly fewer eggs than in water controls. If p-cresol and m-cresol are near each other in neighboring breeding sites, they have a repelling effect on gravid *Ae. aegypti* mosquitoes. In concentration-choice tests, both cresols were deterrents only at the highest concentration.

Organic detritus forms the base of the aquatic food web. Detritus such as dead larvae and decaying plant matter supports microbial growth, making these sites more attractive to gravid females. Organic infusions of fermenting leaves and other plant matter are also significant cues for oviposition. Different leaf infusions can have attractive effects. For instance, hay infusions that contain 3-methylindole are attractive to *Ae. aegypti* (Ganesan et al. 2006). In laboratory settings, sites with fermenting leaf infusions, such as those from white oak and bamboo, also attract more gravid mosquitoes to deposit eggs than sites containing filtered water alone (Ponnusamy et al. 2008). Microbial fermentation of this matter produces kairomones, chemical signals highly attractive to gravid *Ae. aegypti*, which act as oviposition stimulants (Ponnusamy et al. 2008). When these infusions are sterilized, they lose much of their attractiveness for egg laying, as microbial activity is removed; however, they can still lure gravid females to the site (Ponnusamy et al. 2008).

Bacteria are important as they play critical roles in larval development by aiding in energy storage and the biosynthesis of vitamin B9 as well as acting as a source of raw nutrients (Romoli et al. 2021). Aquatic microbial communities produce a variety of oviposition cues. *Pseudomonas aeruginosa* produces decanoic acid, which also serves as a cue for egg deposition (Benzon and Apperson 1988). The bacterial species *Acinetobacter calcoaceticus*, has also been implicated in the selection of oviposition sites. In laboratory studies, water containing larvae and *Acinetobacter calcoaceticus* attracted egg-laying females, whereas water containing only larvae acted as a repellent (Benzon and Apperson 1988). Bacteria produce a mix of carboxylic acids, ranging from nonanoic acid to octadecanoic acid, along with several carboxylic acid methyl esters that can affect oviposition choice (Ponnusamy et al. 2008). Compounds such as nonanoic acid, tetradecanoic acid, and methyl tetradecanoate promote egg laying at low concentrations but deter it at higher doses (Ponnusamy et al. 2008). A 10 ng blend containing 16% nonanoic acid, 83% tetradecanoic acid, and 1% methyl tetradecanoate was highly stimulatory for egg deposition; these ratios were based on profiles from lyophilized bacteria (Ponnusamy et al. 2008). Hexadecanoic acid methyl ester, on the other hand, acts as a repellent, and high bacterial densities in infusions can similarly deter oviposition. These bacterial cues thus provide gravid mosquitoes with nuanced information about habitat quality.

Bacteria can produce VOCs that stimulate oviposition (Ponnusamy et al. 2008). For instance, *Klebsiella sp.* and *Pseudomonas aeruginosa* produce VOCs that elicit contact stimulation or strong antennal responses in gravid *Ae. aegypti* and stimulated oviposition (Benzon and Apperson 1988, Mosquera et al. 2023). These VOCs often act in a dose-dependent manner, with low concentrations promoting oviposition, and higher concentrations acting as repellents (Seenivasagan et al. 2009, Boullis et al. 2021). Experimental work with a binary blend of 2-ethyl-1-hexanol and 2,4-di-tert-butylphenol produced by *Klebsiella sp.* is detected by gravid females’ antennae and elicits a dose-dependent oviposition preference (Mosquera et al. 2023). This binary blend successfully recapitulated the egg-laying responses of gravid mosquitoes. When these cues were tested in an *orco* mutant strain of *Ae. aegypti*, which lacks a functional odorant receptor coreceptor, the mosquitoes failed to respond both behaviorally and physiologically. This finding highlights the critical role of the odorant receptor pathway in mediating chemical communication during oviposition-site selection. This may be because gravid females introduce these bacteria to aquatic sites during egg laying, making the presence of the bacteria and their volatiles a reliable indicator of viable breeding sites to other conspecific females.

Carboxylic acid compounds also serve as cues for oviposition choice and are produced by *Ae. aegypti* larvae, with the highest production in the fourth instar (Wang et al. 2019). Dodecanoic acid (C_12_) and tetradecanoic acid (C_14_) isomers are found in egg cuticles and in all larval and pupae stages. Other egg- and larva-associated compounds include (Z)-9-hexadecenoic acid (C_16_) and octadecanoic acid (C_18_). The egg cuticle also contains fatty acids of carbon chain-length C_12_ to C_1_ as well as methyl esters (methyl dodecanoate, methyl tetradecanoate, methyl (Z)-9-hexadecenoate, methyl hexadecanoate, hexadecanoic acid, methyl (Z)-9-octadecenoate, methyl octadecanoate, (Z)-9-octadecenoic acid) along with a lactone, 6-hexanolactone (Ganesan et al. 2006). Dodecanoic acid and (Z)-9-hexadecenoic acid show positive oviposition responses at 10–100 ppm, with (Z)-9-hexadecenoic acid peaking in attractiveness at 1 to 10 ppm and remaining constant up to 100 ppm (Ganesan et al. 2006). Tetradecanoic acid is most attractive at 1 and 10 ppm but loses effectiveness at higher concentrations, while octadecanoic acid induces a positive response at one ppm and a negative response at higher concentrations (Ganesan et al. 2006).

The straight-chain hydrocarbon n-heneicosane (C21), found on late-stage *Ae. aegypti* larvae cuticles and *Ae. albopictus* larvae, acts as an attractant at 10^−6^ to 10^−5^ g odor plumes or 10 ppm solutions, but repels at concentrations such as 10^−^³ g, which likely indicates an overcrowded site (Seenivasagan et al. 2009, Gonzalez et al. 2016).

Other compounds, such as isopropyl esters, dodecyl lactone, octadecenoic acid, and medium-chain fatty acids, increase in fourth instar larvae (Wang et al. 2019). Isovaleric acid, myristoleic acid, myristic acid, and pentadecanoic acid are found in the fourth instar. Pupae were found to be associated with myristoleic acid, myristic acid, and pentadecanoic acid. Isovaleric acid is also present in larval food infusions and human sweat, indicating bacterial activity and elicits antennal responses from *Ae. aegypti* (Boullis et al. 2021). At 10 ppm, pentadecanoic acid acts as a positive oviposition stimulation cue. At concentrations of 10 to 100 ppm, myristoleic acid and isovaleric acid deter oviposition. A blend of isovaleric acid, tetradecanoic acid, myristoleic acid, and pentadecanoic acid at 1 ppm acts as an attractant, while higher concentrations act as a deterrent (Boullis et al. 2021).

C_9_ acids induce signiﬁcant negative oviposition responses in *Ae. aegypti* at lower concentrations. When the ester concentration increases from 1 to 100 ppm, the ovipositional deterrent effect increases in all cases except for an ester of a saturated C_18_ acid. In addition, methyl octadecanoate acts as a repellent at 10 ppm (Ganesan et al. 2006). The esters contained in eggs may act as oviposition deterrents to prevent conspecific overcrowding, a phenomenon observed in lepidopteran and other dipteran species as well. Higher concentrations appear to be associated with higher egg densities, resulting in a repellent effect.

Across all these chemical classes, a consistent pattern emerges: low concentrations indicate a productive, lightly used habitat, while high concentrations signal overcrowding, decay, or competition. This concentration-dependent switch in behavioral responses highlights how sensitive *Ae. aegypti* females are to nuanced chemical cues. Understanding these cues could guide the development of attract-and-kill ovitraps and other lure-based surveillance tools that exploit the mosquito’s finely tuned chemosensory system.

## Geosmin and Cyanobacteria

Geosmin is a microbial metabolite responsible for the characteristic earthy or musty odor found in surface water, soil, and certain foods (Wnorowski 1992, Liato and Aïder 2017). For humans, geosmin is typically perceived as an unpleasant odor and is resistant to removal by common water purification methods, such as chlorination or oxidation (Wnorowski 1992). Its synthesis involves the methylation of L-methionine and folic acid (Wnorowski 1992). Cyanobacteria, such as *Fischerella muscicola*, produce the highest geosmin concentrations during their lag phase, with yields decreasing as the bacterial culture ages (Wnorowski 1992). Geosmin can be produced under both aerobic and anaerobic conditions (Wnorowski 1992).

The odors present in water bodies depend on the type and bloom of cyanobacterial species, with intensity and chemical profile varying accordingly (Lee et al. 2017). Different types of cyanobacteria produce either toxins or geosmin, serving as primary producers in both aquatic and terrestrial habitats (Churro et al. 2020). Cyanobacteria, such as *Oscillatoria simplicissima* and *Anabaena scheremetievi*, are notable producers of geosmin in water (Lee et al. 2017). The gene coding for the enzyme responsible for geosmin production is found across cyanobacteria, actinobacteria, and delta- and Gammaproteo­bacteria, and it exhibits high sequence similarity among soil and freshwater strains (Churro et al. 2020).

Cyanobacteria can be found throughout the water column with some species localized to the surface of the water, some migrate to different depths in response to environmental stimuli, and others are found primarily in the sediment (Carratalà et al. 2023). Cyanobacteria regulate their depth through a combination of buoyant gas containing vesicles and use carbohydrates as ballast to sink; with water column depth preferences varying depending on species (Carratalà et al. 2023). These bacteria are accessible to larvae and may provide nutritional benefits (Vázquez-martínez et al. 2002, Carratalà et al. 2023). Cyanobacteria are a key food source for some mosquito larvae, including *Anopheles albimanus Wiedemann* (Vázquez-martínez et al. 2002). In southern Mexico, cyanobacteria were prevalent in larval habitats, particularly in permanent freshwater bodies (Vázquez-martínez et al. 2002). *Ae. aegypti* larvae have been documented feeding on cyanobacteria (Garcia-Sánchez et al. 2017). It is hypothesized that *Ae. aegypti* larvae associate the presence of geosmin with cyanobacteria, which signals the presence of a primary food source (Vázquez-martínez et al. 2002).

Environmental factors can modulate geosmin production; higher nitrogen-to-phosphorus ratios limit geosmin synthesis by cyanobacteria (Lee et al. 2017). Ozone treatment can reduce geosmin odors, replacing them with a fruity odor instead (Lee et al. 2017). *Gravid Ae. aegypti* correlates the presence of geosmin with the presence of microbes, a larval food source, in the water, and thus choose to lay their eggs in that site (Melo et al. 2020). In vector control applications, geosmin has been successfully used as bait in oviposition traps to capture gravid *Ae. aegypti* females (Melo et al. 2020). Beetroot peel can serve as a natural substitute for geosmin in such traps (Melo et al. 2020).

## Impact of Larval Nutrition on Adult Behavior

There can be many factors, including genetic and environmental factors, as well as factors related to pathogen populations, that determine mosquitoes’ capacity to serve as effective disease vectors (Carvajal-Lago et al. 2021). A key predictor of an adult mosquito’s ability to serve as a disease vector is the nutrition of its larvae.

The quality of nutrition available to *Ae. aegypti* larvae can significantly shape various aspects of adult behavior and physiology. Well-nourished larvae typically develop into larger, more robust adults with higher fecundity, greater energy reserves, longer lifespans, and increased success in blood feeding (Klowden et al. 1988, Carvajal-Lago et al. 2021). Better energy reserves retained from their larval stage enable the adult to obtain its initial meal and increase survivability (Carvajal-Lago et al. 2021). These adults are better equipped to fight parasites and avoid infection. In contrast, larvae that experience poor nutrition due to lack of food or the necessity of rapid development in an ephemeral water source, grow into smaller adults, which may require 2 blood meals to produce a single batch of eggs (Klowden et al. 1988). Smaller adults are also less inclined to immediately seek hosts for blood meals post-eclosion and/or have reduced ability to do so. As such they may prioritize nectar feeding to provide additional energy for flight and developmental processes (Klowden et al. 1988, Carvajal-Lago et al. 2021). These mosquitoes may have increased susceptibility to arbovirus infection as adults because, as larvae, limited nutritional resources were allocated to development and survival rather than to immunity (Carvajal-Lago et al. 2021). For instance, with the Sindbis virus, adults that had poor nutrition as larvae had a higher infection rate than those with proper nutrition (Carvajal-Lago et al. 2021). Also, *Ae. aegypti* adults derived from larvae that had low-insect detritus related to a high nitrogen percentage presented the highest rates of infection with the Zika virus (Carvajal-Lago et al. 2021). However, the data from lab-based experiments on Sindbis virus and Zika virus may not entirely reflect real-world conditions due to the complexity of ecological interactions and physiological states. These factors could incorporate additional variables that could complicate our understanding of the true relationship between larval nutrition and adult vectorial capacity. A longer lifespan can also allow an infected mosquito to infect more hosts in its quest for blood meals for egg production. There are different effects regarding poor larval nutrition and the ability to vector different diseases as adults (Carvajal-Lago et al. 2021).

Host-seeking behavior in female *Ae. aegypti* is further modulated by access to sugar after blood feeding. When provided with sucrose after a blood meal, females reduce their host-seeking activity as they develop their eggs. However, if sucrose access is restricted, they maintain active host-seeking behavior (Klowden et al. 1988). Competition among larvae during development can also influence adult reproductive ability, suggesting that increased competition may lead to less successful reproduction in emerging adults (Klowden et al. 1988).

In laboratory studies, larvae provided with ample nutrition develop synchronously and produce large, healthy adults (Klowden and Lea 1978). Larvae fed poor diets emerge as smaller adults at staggered times (Klowden and Lea 1978). Field-collected adults are typically smaller than laboratory-reared ones, often requiring 2 blood meals to complete one reproductive cycle (Chadee 2012).

Blood meal size in adult females is a critical factor in determining reproductive success and the cessation of host-seeking behavior. Depending on the size of the blood meal and the adult mosquito, the amount of blood may be sufficient to produce a batch of eggs and may cause enough abdominal distension to signal the female to cease host-seeking activities (Klowden and Lea 1978).

A well-fed larva that develops into a robust female will require between 2.6 and 3.5 μl of blood to stop seeking hosts. If the blood meal exceeds 4 μl, host-seeking behavior ceases entirely. Smaller-sized females require less blood (1.6 to 2.5 μl) to suppress host-seeking behavior, underscoring the relationship between blood meal volume and adult mosquito size (Klowden and Lea 1978). Additionally, the density of mosquitoes feeding on a host can affect blood meal size, as high densities may make hosts more defensive, leading to smaller blood meals and the need to feed again (Klowden and Lea 1978).

## Knowledge Gaps and Application of New Technologies to Old Questions

Considerable research progress in this field has identified an array of chemosensory cues that mediate *Ae. aegypti* oviposition site selection. However, many knowledge gaps remain to be explored to deepen and add nuance to our current understanding. These gaps are in part due to the experimental difficulties of controlling complex multivariate systems while maintaining fidelity with field conditions. One of these challenges comes from the use of long colonized strains of *Ae. aegypti*, such as the Rockefeller strain which has been in culture for decades and lacks the myriad genetic adaptations and environmental cues that guide the behavior of wild mosquitoes. These experiments are often performed in small cages that cannot completely reflect environmental contexts. This leaves open whether identified attractants, repellents, stimulants, and deterrents retain their behavioral activity for wild-type populations navigating complex 3-dimensional environments with competing odor plumes and multiple candidate sites. Cue validation using recently colonized populations under semi-field or full-field conditions is important for translation of laboratory findings into vector surveillance and ­control tools.

Another challenge faced by researchers is the development of ways to integrate the effects of crosstalk across multiple sensory modalities including vision, olfaction, gustation, and mechanosensory stimuli. The question of how different stimuli are weighted, integrated, and acted on during decisions on whether a site would be a good habitat for offspring is important to understand, but also technically difficult. However, this knowledge would be extremely informative in understanding how different stimuli should be integrated into ovitraps to optimize their function as well as improving our understanding of the ecological dynamics site choice in urban and sub-urban environments.

Additional investigation would benefit the development of a deeper understanding of the role inorganic water chemistry plays in site preference. The role of sodium concentration on site selection by *Ae. aegypti* is fairly well understood. However, there is relatively little information available on the influence of other ions and minerals such as ammonium, phosphate, calcium, and magnesium. More information on the role these ions play in site selection in terms of dose response, effects on volatile organic compounds, and dynamics of microbial communities at different concentrations could provide important understandings of the role of water chemistry on chemosensory stimuli.

Finally, while microbial communities are known to be important for the production of chemosensory cues and as a source of nutrients for developing larvae, the field lacks information on the metabolic functions provided by the various microbial taxa found in oviposition sites and how these functions interact with environmental substrates to produce chemosensory cues. Identification of key microbial constituents or specific metabolic functions required for production of chemosensory products would also provide key information for production of standardized and deployable microbial lures for oviposition trap optimization.

These are all difficult subject areas to approach. However, the development of new high throughput technologies such as metabolomic analyses, metagenomic sequencing, new spectral analyses (such as Raman and infrared spectroscopy), and advanced statistical analyses and modeling of complex multivariate datasets allow for detailed investigations of these complex systems. Leveraging these new technologies to expand on and deepen existing understandings could provide novel insights into oviposition biology and significant improvements on existing gravid trap technologies.

## Conclusion: Application in Vector Surveillance and Control

A detailed understanding of *Ae. aegypti* oviposition behavior, including its regulation by VOCs, microbial metabolites, conspecific and heterospecific cues, and environmental context, holds considerable promise for enhancing vector surveillance and control strategies. Ovitraps have proven highly effective for monitoring *Ae. aegypti* populations because they exploit the natural tendency of gravid females to lay eggs in man-made containers (CDC 2020, McGregor and Connelly 2021, Walker et al. 2021, Otero et al. 2025). The traps are useful for surveillance and population control, are typically monitored weekly, and are designed to prevent the emergence of adult mosquitoes. They are cost-effective, easy to move, and are not detrimental to the location where they are placed. Additionally, eggs can be reared to identify the mosquito species present in the area.

However, ovitraps do have some drawbacks that limit their efficacy relative to traps that utilize host cues which target blood meal seeking females. Gravid autocidal traps have some requirements which can complicate the logistics of their implementation. Multiple traps per household are required to compete with natural oviposition sites and source reduction prior to deployment is needed to optimize efficacy. Traps also require regular maintenance to remain operational. These factors require community cooperation and high logistical capacity which can be difficult to maintain in under-resourced areas (Barrera et al. 2014, Acevedo et al. 2016). Beyond the logistical issues, optimization of traps with the right oviposition attractants and stimulants is essential to their function.

In spite of these caveats, gravid autocidal traps remain a promising and environmentally friendly method of vector control which has the potential to be significantly improved via attractant optimization, implementation of public education and outreach efforts, and modification of suburban and urban infrastructure development practices to minimize the presence of human derived breeding sites. Incorporating attractants mentioned in this review, such as p-cresol, 1 ppm of caproic acid, bacterial VOCs, or microbial kairomones, can further enhance ovitrap efficacy and specificity, while adding larvicides, such as granular temephos, an organophosphorus larvicide, creates lethal lures that reduce mosquito populations at the source (Ong and Jaal 2015, Martínez-Mercado et al. 2022).

As *Ae. aegypti*’s range continues to expand due to urbanization and climate change, deploying surveillance and control tools that align with the species’ oviposition ecology will become increasingly important. Continued integration of physiological and behavioral data from laboratory and field studies will support the refinement of mosquito monitoring and ­control. Ultimately, translating oviposition behavior into field-applicable technologies will strengthen proactive vector management and mitigate the risk of arbovirus transmission in both endemic and newly affected regions.
